# Developing and Piloting a Standardized European Protocol for Hepatitis C Prevalence Surveys in the General Population (2016–2019)

**DOI:** 10.3389/fpubh.2021.568524

**Published:** 2021-05-28

**Authors:** Ida Sperle, Stine Nielsen, Viviane Bremer, Martyna Gassowski, Henrikki Brummer-Korvenkontio, Roberto Bruni, Anna Rita Ciccaglione, Elena Kaneva, Kirsi Liitsola, Zlatina Naneva, Tanya Perchemlieva, Enea Spada, Salla E. Toikkanen, Andrew J. Amato-Gauci, Erika Duffell, Ruth Zimmermann

**Affiliations:** ^1^Department of Infectious Disease Epidemiology, Robert Koch Institute, Berlin, Germany; ^2^Charité, Universitätsmedizin Berlin, Berlin, Germany; ^3^Independent consultant, Madrid, Spain; ^4^Department of Health Security, Finnish Institute for Health and Welfare, Helsinki, Finland; ^5^Department of Infectious Disease, Istituto Superiore di Sanità, Rome, Italy; ^6^Regional Health Inspectorate, Stara Zagora, Bulgaria; ^7^European Centre for Disease Prevention and Control, Stockholm, Sweden

**Keywords:** hepatitis C, HCV, general population, prevalence, technical protocol, surveys, questionnaires

## Abstract

**Background:** A robust estimate of the number of people with chronic hepatitis C virus (HCV) infection is essential for an appropriate public health response and for monitoring progress toward the WHO goal of eliminating viral hepatitis. Existing HCV prevalence studies in the European Union (EU)/European Economic Area (EEA) countries are heterogeneous and often of poor quality due to non-probability based sampling methods, small sample sizes and lack of standardization, leading to poor national representativeness. This project aimed to develop and pilot standardized protocols for undertaking nationally representative HCV prevalence surveys in the general adult population.

**Methods:** From 2016 to 2019 a team from the Robert Koch-Institute contracted by the European Centre for Disease Prevention and Control synthesized evidence on existing HCV prevalence surveys and survey methodology and drafted a protocol. The methodological elements of the protocol were piloted and evaluated in Bulgaria, Finland and Italy, and lessons learnt from the pilots were integrated in the final protocol. An international multidisciplinary expert group was consulted regularly.

**Results:** The protocol includes three alternative study approaches: a stand-alone survey; a “nested” survey within an existing health survey; and a retrospective testing survey approach. A decision algorithm advising which approach to use was developed. The protocol was piloted and finalized covering minimum and gold standards for all steps to be implemented from sampling, data protection and ethical issues, recruitment, specimen collection and laboratory testing options, staff training, data management and analysis and budget considerations. Through piloting, the survey approaches were effectively implemented to produce HCV prevalence estimates and the pilots highlighted the strengths and limitations of each approach and key lessons learnt were used to improve the protocol.

**Conclusions:** An evidence-based protocol for undertaking HCV prevalence serosurveys in the general population reflecting the different needs, resources and epidemiological situations has been developed, effectively implemented and refined through piloting. This technical guidance supports EU/EEA countries in their efforts to estimate their national hepatitis C burden as part of monitoring progress toward the elimination targets.

## Background

The World Health Organization (WHO) has set ambitious targets for the elimination of viral hepatitis as a public health threat by 2030 in the global health sector strategy on viral hepatitis 2016–2021 (1).

One of the five strategic directions outlined in the strategy entails information for focused action, underlining the importance of collecting robust data on the viral hepatitis epidemic in order to improve and guide implementation of efforts in the response. An update on the progress of the implementation of the strategy was recently published by WHO, stressing the need to strengthen and more regularly update viral hepatitis data in order to improve implementation (2). Robust estimates of the number of people with chronic hepatitis C virus (HCV) infection are needed and its prevalence is one of 10 core indicators (C.1.b), identified by the WHO in their framework on monitoring and evaluation for viral hepatitis (3).

Data on newly diagnosed and notified cases of viral hepatitis are collected through the surveillance systems, which are in place for HCV in the majority of countries in the European Union (EU). However, completeness of data is a major issue, and reporting of data according to EU case definitions to enable a clear comparison across countries and time remains challenging (4, 5). Furthermore, the data collected through the surveillance systems are largely influenced by the local testing strategies rather than actual epidemiological trends or burden of disease.

HCV prevalence surveys provide key information on the epidemiology of HCV infection. These surveys, in contrast to surveillance data, provide a snapshot of the current epidemiological situation, as all individuals in the sample infected with HCV are identified, regardless of their diagnostic status. However, a recent systematic review found that up-to-date estimates of prevalence are lacking from many EU/European Economic Area (EEA) countries (5, 6). This review also found that studies that have been undertaken in the EU/EEA are heterogeneous and often of poor quality due to non-probability based sampling methods, small sample sizes and lack of standardization leading to poor national representativeness (5, 6).

The HCV epidemiology varies between countries and depends on multiple factors. In countries with low prevalence, injecting drug use (IDU) is an important risk factor and a main contributor to the HCV epidemic (7). In these countries, people who inject drugs (PWID) are often the group with the highest prevalence and a key population to target with prevention and treatment measures. In other countries, where higher levels of transmission occurred in the past through unsafe injections, via blood transfusions or other nosocomial transmission routes such as unsafe use of glass syringes, as reported in Italy (8), HCV is more widespread in the older general population (9). This type of more generalized epidemics has been observed in some European countries such as Czechia, Italy, Poland and Romania (10–14).

Knowing the HCV prevalence in the general population, and standardizing the way data are collected and estimates generated will contribute to more robust data allowing monitoring and comparisons between countries and over time (15). This will positively contribute to the monitoring and tracking of the progress toward the WHO viral hepatitis elimination goal (3).

To address this issue and support EU/EEA Member States (MS) in their efforts to generate robust estimates of HCV prevalence, the European Center for Disease Prevention and Control (ECDC) launched the “Sero-Prevalence Survey for Hepatitis C in Europe” (SPHERE-C) project. The Robert Koch Institute (RKI) was formally contracted by ECDC between 2016 and 2019 to develop a detailed technical protocol, with the aim to develop and pilot standardized protocols for undertaking nationally representative prevalence surveys of HCV in the general adult population (15).

## Methods

A short inquiry was sent to all ECDC national focal points for hepatitis in the EU/EEA MS in September 2016 to gain insight in the countries' availability of HCV prevalence data from previous surveys and around future plans for undertaking work in this area, as well as gauging interest in participating in a pilot of the SPHERE-C protocol in 2018. Responses from 22 MS were obtained and used to guide the development of the protocol.

The development of the protocol was based on synthesis of scientific information and evidence on HCV prevalence surveys. A desktop review was conducted to define all the objectives for the survey and to suggest methods for each objective. To inform these objectives, a literature review was undertaken to gain understanding of the local epidemiological gaps and political needs. Thereafter, to identify the most appropriate methods for the defined objectives, available information on the methods used in previously conducted HCV prevalence surveys was collected, and efforts were made to also identify surveys outside the EU/EEA. The identified surveys and key information were entered into a table, and study protocols were collected through online searches or through contact with the researchers who performed the surveys. Methodological criteria to achieve minimum or gold standard for each objective was identified and a conceptual matrix presenting the findings was constructed with areas covering selection of sites/population, sampling and stratified sampling methods, specimen/data collection, laboratory testing methods, storage and transport of samples, confidentiality and ethical issues, data management, quality control and training materials needed.

An expert group was set up to guide the direction of the project and to provide feedback to the development of the protocol. The expert group consisted of researchers, laboratory experts, statisticians, medical doctors and epidemiologists from across Europe and the USA. Three face-to-face consultations were held with the expert group between 2016 and 2019. The group was asked to comment on draft versions of the protocol over the course of the project. The expert group agreed upon the most relevant methodological approaches to be included in the protocol based on the evidence presented by the RKI project team and through consensus.

Three EU countries were selected to pilot the technical protocol. Methodological elements in the protocol were piloted to gather practical experience and evaluate its usability and applicability. Lessons learnt were collected to guide the further development of the protocol.

The following three pilots were carried out during 2018:

- A retrospective survey with testing of blood samples from the FinHealth2017 national health examination survey in Finland
- A stand-alone survey in the city of Stara Zagora, Bulgaria
- A stand-alone survey in the city of Catanzaro, Italy

A pilot-specific study protocol based on the overall protocol and study materials were developed for the pilot of the stand-alone survey conducted in Stara Zagora, Bulgaria by the RKI. The local survey teams in Finland and Italy developed their own pilot-protocols and materials, based on the recommendations from the technical SPHERE-C protocol. The aim for each of the three surveys was formulated and tailored to the local context drawing on the recommended aim in the technical protocol. All three pilots were performed in close collaboration with the team at RKI, and regular teleconferences were held with the local survey teams to ensure that decisions made locally were coherent with the technical protocol.

Indicators were developed to evaluate the feasibility of the protocol and the methodological approaches. The evaluation indicators were transformed into an evaluation questionnaire with 10 main questions covering all sections in the technical protocol including objectives of the survey, sampling and sample frame, time spent, structure, coordination and collaboration, ethical approval, data protection and informed consent, awareness-raising, recruitment, personnel, budget, data management and data collection (blood sampling and questionnaire). The evaluation questionnaire was completed in writing by the local survey teams in the three countries, and then sent electronically to the RKI. Interviews to explore issues in further depth were conducted with the local survey teams on the phone with the survey teams from Finland and Italy, and face to face during a 2 day evaluation workshop in December 2018, at the RKI in Berlin, Germany with the survey team from Bulgaria.

## Results

The technical protocol provides background as well as more detailed information demonstrating the importance of undertaking prevalence surveys to generate robust estimates of hepatitis C prevalence. Importantly, it provides options and steps for planning and conducting a population-based hepatitis C survey which can be adapted to the local context. The technical protocol consists of two main parts:

1) Selection of a survey approach
2) Planning and conducting a survey

This is explained in detail in the published protocol (15), and in brief below.

### Three Survey Approaches

The technical protocol includes three survey approaches which were identified as the best approaches through the desktop review and through discussions with the expert group. The three survey approaches are: a survey “nested” within an upcoming health survey; a retrospective testing survey; and a stand-alone survey.

The three survey approaches all fulfill the pre-defined criteria outlined in the protocol and are variations of a survey with probability-based sampling. The protocol covers minimum and gold standards for key aspects including: sampling; data protection; ethical issues; recruitment; specimen collection; laboratory testing; staff training; data management; quality assurance and budget considerations (15). As an example, for the type of specimen, the minimum requirement is dried blood spots, and the gold standard is venous blood samples (15).

**Figure 1:**
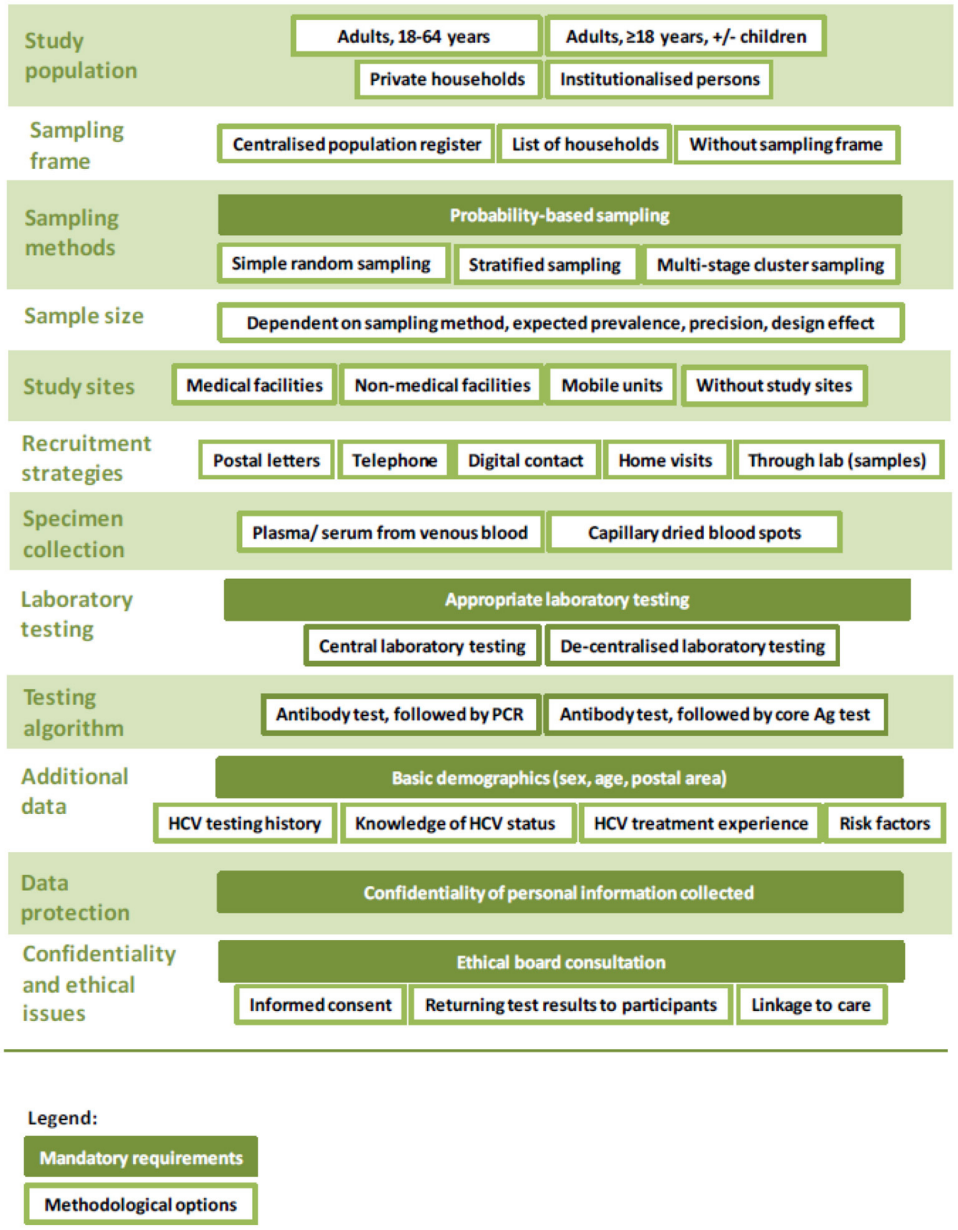
Overview of mandatory requirements and methodological options for an HCV prevalence survey.

Mandatory requirements and methodological options for an HCV prevalence survey (for all three survey approaches) are illustrated in Figure 1 and described in more details in the published protocol (15).

#### Nested Survey

The nested survey approach requires an upcoming larger population-based health survey of the general population, e.g., a national health examination survey (HES). In this approach, the prevalence survey is nested in this larger survey, which makes it less resource intensive and costly due to the use of the existing infrastructure of the already planned survey. This allows additional testing of the participants for HCV, as well as collection of HCV-related behavioral data, with little extra effort. Therefore, this option requires relatively small amounts of financial and human resources. The chances of a representative sample are increased if the sample size calculations for the HES are sufficient for the expected prevalence of HCV due to the often rigorous sampling strategy and efforts to reduce non-response, that are part of a larger population-based survey.

#### Retrospective Testing Survey

This approach requires a recently conducted population-based survey. From stored blood samples of a former survey, HCV testing can be performed retrospectively. The criterion of probability-based sampling needs to be fulfilled. Furthermore, it is important to ensure that there is a sufficient number of samples with enough material left for testing, and that these do not represent a biased sub-set of the original samples collected. Further, informed consent that was given by participants needs to include storage of samples for further research and retrospective testing. If the abovementioned requirements are fulfilled, extra costs for this approach will mainly arise from the laboratory work and analysis of the data.

#### Stand-Alone Survey

The third option is to embark on a stand-alone HCV prevalence survey where the primary aim is to estimate the HCV prevalence (by age and sex). This is the most staff- and financial resource intensive approach, as all steps needed to do a survey, including sampling, data protection and ethical issues, recruitment, specimen collection and laboratory testing options, staff training, data management and budget considerations, need to be performed.

### Selecting a Survey Approach

**Figure 2:**
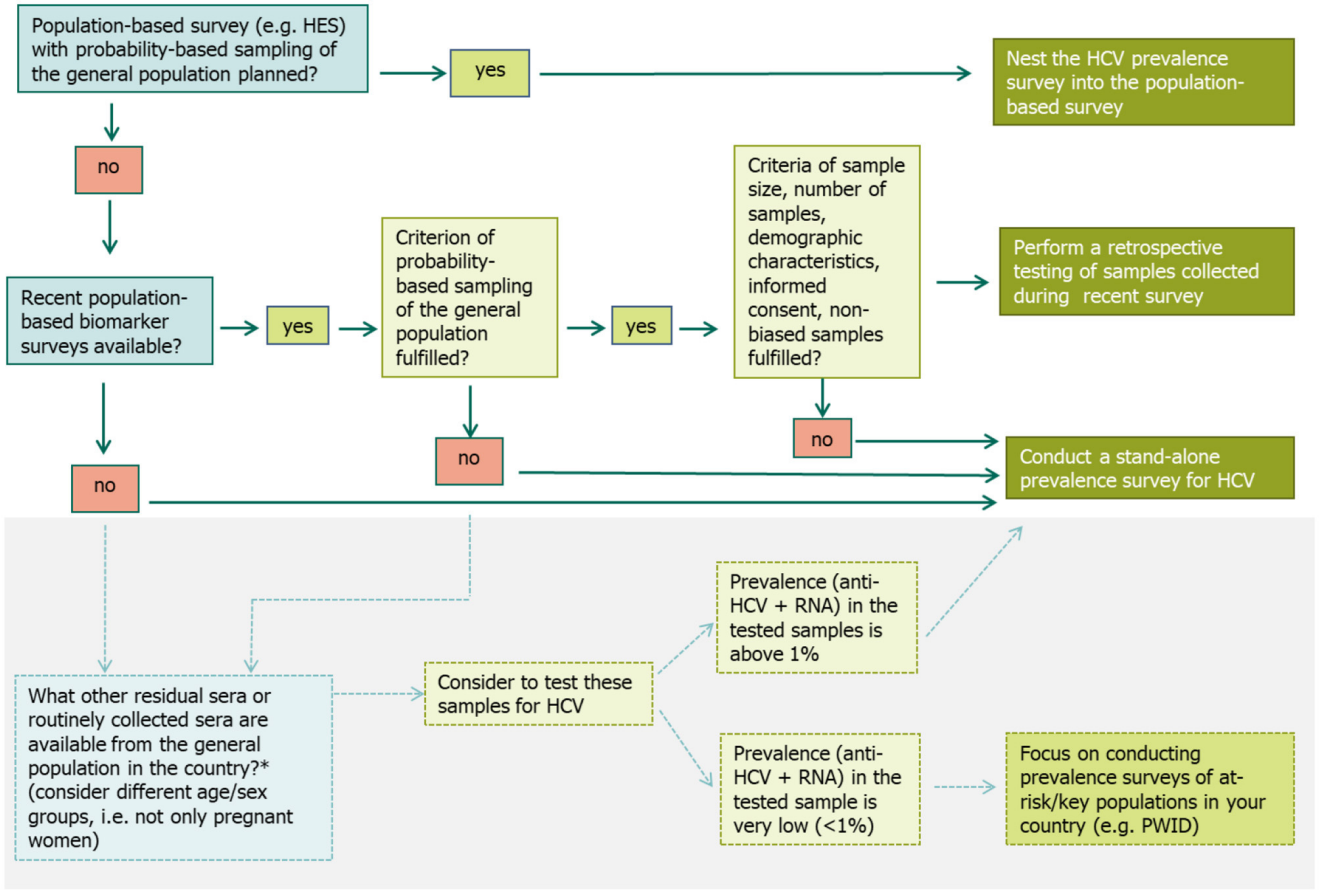
Decision algorithm to select the most suitable survey approach when planning a prevalence survey for hepatitis C in the general population (15). *Alternative options exist that might be explored by countries to get an idea of the HCV prevalence level in the general population, if they do not have data from a recent population-based prevalence survey or plans for a future survey and few resources for a stand-alone survey (15).

A decision algorithm was developed and included in the protocol to guide MS through a careful decision making process when selecting the most suitable survey approach for their respective setting and situation (Figure 2) (15).

If a large population-based survey is planned, where blood samples are collected (e.g., a HES with a probability-based sampling of the general population), it is suggested to nest the HCV prevalence survey into this population survey. The precondition is that the planned survey fulfills the minimum criteria outlined in the protocol, e.g., has a sufficiently large sample size and is representative of the populations of interest. Including HCV testing in existing survey protocols involves steps similar to those for designing a new survey, although some steps may be simpler as they have already been done for the original survey (such as ethical approval, sampling process, and the recruitment strategy).

If no population-based survey is planned, but a former survey such as a HES or another study with a probability-based sample of the general population was conducted recently and included the collection of blood samples, an option is to test the sera left over from this survey retrospectively. Again, the above mentioned criteria need to be met to ensure the quality of the data generated. Furthermore, proper sample storage should be assured to prevent bias due to HCV RNA degradation.

If none of the two above options are available, then a third option is to do a stand-alone survey, where the primary purpose is to estimate the HCV prevalence. When conducting a stand-alone survey, all the steps for undertaking a survey need to be carefully planned and undertaken. Setting up a stand-alone survey in the general population is time- and budget intensive. Therefore, a preliminary first step is to test any residual or routinely collected sera (e.g., from antenatal care screening). If the prevalence in those samples is found to be low (<1%), it is recommended that prevalence surveys in key populations at higher risk of infection, e.g., among PWID should be prioritized over a population-based survey in the general population (Figure 2).

If none of the three survey approaches are possible there are several alternative methods to consider, although these methods may be more subject to potential bias. These include testing residual sera from laboratory samples (16, 17), samples from proxy populations of the general population such as pregnant women (18) or first-time blood donors (6) or general practitioner or health insurance registries as well as linking information from multiple national registries and applying various modeling techniques (19, 20). These and more alternative methods are explained in further detail the technical protocol (15).

### Results From Piloting the Protocol

The three separate survey approaches were planned to be piloted in three different EU countries. However, these plans were subsequently adapted on account of the local situation in each of these three sites, so that finally the stand-alone survey approach was piloted in two countries and the retrospective testing approach was piloted in the third country. Furthermore, due to local circumstances the recommended steps in the protocol for the different survey approaches were adapted to fit with what was feasible and in agreement with the local context in the three countries.

### Stand-Alone Survey Approach

#### Stara Zagora, Bulgaria

The main objectives of this pilot were to estimate the prevalence of chronic HCV infection, by sex and age group, in the adult population in the city of Stara Zagora, Bulgaria and to test the feasibility and proposed methodological approach in the draft technical SPHERE-C protocol.

#### Italy

In Italy, the initial plan was to nest the HCV survey onto a planned HES focused on salt consumption (CUORE^1^). However, this needed to be adapted as the sample size in the CUORE survey was too small. Therefore, the sample size was re-calculated and the local team took the decision to undertake a stand-alone survey.

The objectives of the survey pilot in Italy were to estimate the age- and sex specific prevalence of chronic HCV infection, age- and sex specific prevalence of exposure to HCV and the prevalence of undiagnosed HCV in the adult population of the city of Catanzaro, Southern Italy. All these objectives were fulfilled.

### Nested Survey Approach With Retrospective Testing of Samples

#### Finland

The main objectives were to estimate the anti-HCV and prevalence of chronic infection in the Finnish general population (above 18 years of age) using the samples from the FinHealth2017 national health examination survey. A secondary objective was to match the data with the national infectious disease register, in order to generate an estimate of the undiagnosed fraction. The objectives of the survey were fulfilled.

### General Results

From the evaluation of the pilots and the technical protocol, various challenges were reported by the local survey teams. In Table 1 below, the sections included in the technical protocol are listed together with key lessons learnt from the three pilot surveys, and implications for the protocol. The detailed results of the pilot in Bulgaria are published elsewhere (22).

**Table 1:** Summary of methodological details, results of the pilots, lessons learnt, and implications for the technical protocol (15).

	**Stand-alone survey**		**Nested with retrospective testing of samples**	**Implications for protocol**
	**Bulgaria**	**Italy**	**Finland**	
Data protection issues/Ethical approval	Names and addresses of invitees were not allowed to be shared with study team, invitation letters needed to be sent out by the municipality holding the register	Required to call every participant for scheduling appointment to return test results	Data protection issues and ethical approval conducted previously by FinHealth study team. Informed consent form already included possibility of testing for some other diseases	Plan for getting the ethical approval early to be able to still adjust according to requested changes Data collection and processing according to the General Data Protection Regulation (GDPR) in the EU 2016/679 required, therefore early contact with the national data protection agency advised
Sampling method	Simple random sample stratified by age and sex	Simple random sample stratified by age and sex	Two-stage cluster sampling stratified by age and sex	Sample should be selected using a probability-based random sampling method For smaller geographical areas (e.g., cities) simple random sampling may be applied
Sampling frame	Local population register of the city of Stara Zagora	Local population register of the city of Catanzaro	National population register	Population registers should be up to date
Sample size calculation	*N* = 999 (expected prevalence of chronic HCV was 1.0% and a lower precision bound of 0.25%)	*N* = 889 (expected prevalence of of chronic HCV infection of 1.0% for age group 35–65 (upper precision bound 2.2%) and 5.0% for age group 65+ (upper precision bound 10.0%)	*N* = 10,305 (expected prevalence of current HCV infection (anti-HCV and HCV RNA positive) of 1% and a lower precision bound of 0.25%)	Ensure large enough sample size to get a valid estimate [Input and statistical formula on how to calculate sample size included in technical protocol (15)]
Recruitment strategy	Tracked invitation letter. Reminders: a second tracked invitation letter	One invitation letter (in 4 rounds). For each round a new subset of the sample was invited	First contact with a postcard, followed by an invitation letter. Reminders: postcards, phone calls, SMS reminders	Emphasize that more recruitment efforts are needed to ensure a high enough response rate and to include the “hard to reach” populations who may have a poorer health Only tracked letters are not recommended Make at least three attempts to reach participant (invitation letter, reminder letter, phone call, SMS reminders, or house visits) Include a pre-test to test the effectiveness of different incentives
Promotion of the survey	Information leaflet for invitees; contact with and engagement of local authorities; local media campaign to inform about hepatitis C and encourage participation in the survey including information posters in local pharmacies and outpatient care facilities (general practitioners and medical centers); 3 local press conferences, local radio and television broadcasts	Information leaflet for invitees; contact with and engagement of local authorities; awareness posters for the survey displayed in waiting rooms of general practitioner practices and in the hospital of Catanzaro	Information leaflet for invitees; contact with and engagement of local authorities; Press conference, newspaper articles, radio and television broadcasts	Information leaflet (and website) to inform invitees are strongly recommended for all surveys Information and promotion of the survey among the general population through media and local authorities, and among health care staff are recommended
Data collection period	10 weeks (5 September 2018–16 November 2018)	4 rounds of 1 week each in a period of 7 months(June 2018–December 2018)	7 months (January 2017–July 2017)	Plan extendible data collection period/buffer of time in case sample size was not reached in the planned period. The data collection period in Bulgaria should have been prolonged to reach sample size
People invited	1,998 (1,166 picked up their letter)	9,000 (8,655 letters delivered)	10,247	Take into account expected non-response rate, and consider that the non-response rate may be higher than 50%
Participants	252	1,003	5,923 available samples tested	
Incentives	A coffee mug and a pencil	One day off from work for participants	Results of the health examinations and laboratory analysis of the collected biological samples	Consider different incentives for different age-groups. Include a pre-test to test the effectiveness of different incentives
Response rate	12.6% Net response rate: 21.6% (of those who got the invitation)	11.1% Net response rate: 11.6% (of those who got the invitation)	Overall response rate for questionnaire: 59.6% Net response rate for health examination: 57.8%.	Low response rates in all pilots highlight the challenge of reaching the target set by EHES of 70% (15, 21) and consideration of a more realistic target
Additional data and questionnaire	Self-administered questionnaire including questions specific to HCV Migrants were not sufficiently included, and therefore unknown if translation was needed	Self-administered questionnaire including questions specific to HCV Migrants were not sufficiently included, and therefore unknown if translation was needed	Self-administered questionnaire completed before HES either electronically or manually No HCV-specific questions (e.g., HCV infection risks) included	Self-administered questionnaires work well in general populations Prior to data collection, assess whether translation /interviews are needed In nested surveys, early collaboration with survey team important to ensure that HCV-related questions are included
Laboratory	Local laboratory for serology and one in capital for confirmatory testing and PCR. Shipping by using routine procedures	Shipping of samples to a centralized reference laboratory for all testing	Defreezing, aliquoting and shipping of samples to another laboratory for serology and PCR	Centralized testing of all steps in one laboratory is recommended Alternatively two-step test algorithm in two laboratories when routine shipping procedures can be used In retrospective design, samples for HCV testing should be aliquoted during data collection
Testing algorithm	Anti HCV ELISA, followed by PCR. Immunoblot for PCR negative samples	Anti HCV ELISA, followed by PCR. Immunoblot for PCR negative samples	Anti HCV ELISA, followed by Immunoblot (HCV ELISA positives and borderlines) and PCR (Immunoblot positives and borderlines)	The number of false positives may be high in low prevalence settings, therefore confirmation of anti-HCV reactive, PCR negative samples is important in these settings
Returning test results to participants and linkage to care	Test results were returned to all survey participants, who received a letter with their participant ID and a date for when they would receive their test result in person at the Regional Health Inspectorate. Those who were tested positive were linked to specialised medical care	All participants contacted via phone to schedule an appointment during which they would receive their test result Those who were tested positive were linked to specialised medical care	Positive cases were contacted by phone and a letter. Those who were tested positive were linked to specialised medical care	Plan enough time, staff and budget to have appointments with all participants or outsource the scheduling of appointments Alternatively only inform positive-tested about test results Returning test results from retrospective testing only if data was collected recently, and participants consented to being informed
Data analysis including weighting	Frequencies and percentages were calculated for categorical variables (participants and non-participants). For the chronic HCV prevalence weighting adjustment was performed with age and sex. Prevalence estimates were calculated as crude estimates and weighted estimates with 95% Confidence Intervals All analyzes were carried out in Stata 15.1	Non-response biases were evaluated by comparing respondents and non-responders with regard to their sex, age distribution and housing deprivation level. Crude, age and sex specific, and standardized anti-HCV prevalence rates were calculated. The associations of HCV infection with the different predictor variables were investigated by log binomial regressions with sampling weights or by exact logistic regressions as appropriate. Variables with a *p* < 0.20 at the univariate analysis were considered as potential predictors and included in multivariable analysis All analyzes were carried out in Stata 15.1	Post-stratification weights were used to correct the possible for non-response biases by incorporating population distributions of sex, age and other appropriate characteristics into survey estimates Design based weighted overall and age- and sex-stratified estimates of the HCV prevalence and their 95% confidence intervals were calculated The associations of HCV infection with multiple explanatory variables were modeled using logistic regression model with sampling weights. Predictive margins of interests were calculated	95% confidence intervals (taking into account the design of the survey) Weighting should take into account at least age and sex, if possible, further characteristics (e.g., regional or urban–rural distribution, migration status) 95% confidence intervals (taking into account the design of the survey) Weighting should take into account at least age and sex, if possible, further characteristics (e.g., regional or urban–rural distribution, migration status)
Budget implications	Most time and resources spent on administrative challenges	Most resources spent on sending letters and scheduling appointments	Most time spent on preparing samples for testing	Allow adequate time for administration Consider outsourcing the sending of letters/scheduling appointments

## Discussion

The survey approach selected to estimate HCV in the adult general population needs to be carefully considered. Conducting a population-based survey is challenging, resource intensive, requires a good survey infrastructure, and a sufficient number of well-trained staff members. Therefore, the preferred option is to make use of an already planned population-based health survey, or to make use of retrospective testing of already collected samples, providing that requirements are fulfilled to ensure representativeness. However, these approaches also have their limitations, as, for example, nesting a survey onto a pre-planned survey may not fit in with the scope or logistical capacity of the pre-planned survey.

The evaluation of the three pilot surveys indicated that the different survey approaches selected are suitable methodological designs for estimating the anti-HCV and the chronic HCV infection prevalence in the adult general population. Nonetheless, the pilots were associated with several important limitations. The stand-alone surveys were only conducted on city level, and conducting these on national level is likely to be more complex. The nested survey design outlined in the protocol was not fully piloted, as the survey in Finland adapted the approach and retrospectively tested the samples for HCV. Nonetheless, methodological elements in the technical protocol for conducting HCV prevalence surveys has been demonstrated to be a useful and effective tool for EU/EEA MS as expressed by the local survey teams in the qualitative evaluation (15). Importantly, the protocol considers different situations in different settings by assisting countries through careful decisions that need to be made to select the most appropriate survey approach for any given context.

The technical protocol refers to chronic HCV. Having an up to date estimate of chronic HCV is particularly important given the availability of the direct acting antiviral treatments (DAAs) for HCV. It has been demonstrated that increased access to DAAs leads to a decrease in HCV incidence and prevalence (23, 24). Although low, monitoring the HCV burden and estimating the number of people in need of treatment is of critical importance in the response to viral hepatitis.

### Lessons From the Pilots

Although the nested survey approach is the first approach to consider, it is first and foremost critical that the minimum requirements are fulfilled. This was not the case in the pilot in Italy where the CUORE survey was not powered to estimate the HCV prevalence. However, while it would have been possible to nest onto the survey, and then sample additional people for HCV testing to reach the sample size calculated for the HCV prevalence survey, the Italian survey team decided to change survey approach to a stand-alone approach. This approach however required more efforts in terms of organization and time as well as human and financial resources.

The original plan in Finland was a nested survey. However, delay in getting access to the samples for HCV testing meant that it ended up resembling more a retrospective testing approach. Lessons from the retrospective testing of samples in Finland underlined the importance of communication and mutual understanding between the two teams (main survey team and HCV prevalence survey team) in order to keep the timeline for the HES and ensure the testing of samples for HCV. Early and clear communications may also increase the chances of including extra HCV relevant questions in the questionnaire. For the survey in Finland, questions on past or present drug use were not included to keep the questionnaire short. It is important to be able to standardize results across Europe, and therefore important to collect a minimum set of sociodemographic data for each participant, regardless of survey approach. These include information on sex,) at the time of blood sample collection, and a postal or geographical code. The core set of data, as well as recommended questions on HCV testing and status and risk factors, are provided in the technical protocol (15). There are various strengths using the nested approach, but also important limitations. While a significant advantage is the possibility to make use of an established survey including its sampling approach and the associated socio-economic data, the disadvantage is that there may be limited opportunities to influence the sampling strategy and the overall schedule of the survey, which was a barrier for the Finnish pilot.

Another challenge with the nested approach is interest from different research groups with focus on different disease areas. With a probability-based sampling and rigorous recruitment strategy, the samples are considered of high value and can contribute to valuable knowledge for several disease areas. There are often competing proposals and research ideas from different groups, all wanting to include specific questions in the questionnaire, making early planning and prioritization crucial. In the retrospective testing approach in Finland, more time was needed for sample handling. Therefore, the Finnish team recommends to draw specific samples for infectious diseases testing during the HES, as opposed to only one blood sample which then needs to be tested by multiple groups.

It was not possible to pilot all recruitment steps recommended in the technical protocol (letter, phone calls, short message service (SMS) reminders, and house visits). In Italy, only letters were sent in several rounds, and for each round, a new subset of the sample was invited to participate. While the sample size was reached, the recruitment strategy implemented for the Italian survey may have led to a less representative sample as those who take part after one recruitment attempt are easier to reach and thereby likely in better health or more interested or have more time. Additional recruitment steps are needed to reach initial non-responders, who might differ in socioeconomic and other characteristics from those who more easily accept to participate (25–27). Other innovative approaches may help to increase the number of respondents, e.g., by self-sampling or by offering telephone interview (28, 29). Similarly, the low response rate in Bulgaria is likely to have been caused by the change in recruitment strategy which only allowed invitation via letter. Further recruitment steps are needed to ensure a higher response rate such as e.g., phone calls and house visits (27, 30), which could not be piloted. In Finland, SMS reminders have previously proved successful in increasing participation among young invitees (30). Implementing several recruitment steps, as outlined in the SPHERE-C protocol, is important to ensure a high response rate. If unable to implement enough steps to ensure a high response rate, the large efforts needed to conduct a stand-alone survey may be unwarranted as the end sample will not be representative. In which case, a better choice may be an alternative approach for estimating HCV prevalence.

The impact of incentives depends on the context in which they are offered. While the incentive provided in Stara Zagora was well-received (22), different incentives tailored to different age groups may have resulted in a greater response rate. For all surveys, the most efficient incentives and recruitment efforts need to be locally evaluated, e.g., through a pre-test prior to the survey, and decided upon according to context (26).

It may be that neither of the recommended three approaches are an option for some countries. Therefore, if there are no resources available for a stand-alone survey and testing stored samples or samples from a planned survey is not possible, alternatives may be explored. These may include testing residual sera from clinical laboratories, looking at data from first-time blood donors or looking at data from routine screening of pregnant women (15). These possibilities may also be used to get an idea of what the prevalence is before embarking on a stand-alone HCV prevalence survey. Even if such alternative approaches are likely to be based on non-probability-based sampling which increases the risk of bias, they may provide sufficient evidence for focusing future prevalence surveys in at-risk populations. By testing residual sera from different groups, bias can be reduced (16). It is of crucial importance that regardless of approach and method selected, efforts are made to ensure that the minimum requirements outlined in the technical protocol are met to ensure that results are representative and useful for estimating the HCV prevalence.

If a country sets out to do a stand-alone survey, it is highly advisable to include testing for other infectious diseases, such as hepatitis A, B, D, E, HIV, other sexually transmitted infections, in addition to HCV. It may also be relevant, depending on country and context, to consider including vaccine preventable diseases or relevant non-communicable diseases. A lot of work needs to be put into the planning and conducting of a stand-alone prevalence survey, especially if recommended approaches are taken to ensure a good response rate, and therefore it will make sense to make use of the rigorous sampling strategy to test for other infectious diseases.

### Moving From HCV Prevalence Estimate in General Population to National Prevalence Estimate

Estimating the HCV prevalence in the general population is only one part of getting a national estimate of the HCV prevalence, which is one of the WHO core indicators in the monitoring and evaluation framework (3).

More data and additional methodological approaches are needed in order to generate a national prevalence estimate. Some countries have combined data from multiple registers and applied various modeling techniques to generate national HCV prevalence estimates (20, 31). Others have applied the workbook method (32) or the Bayesian multi-parameter evidence synthesis (MPES) (33). For these approaches, additional activities beyond what is covered in the technical protocol are needed. These activities include identifying the at-risk groups for HCV, which include PWID (both current and former), prison population, men who have sex with men (MSM) and migrants (documented and undocumented), then estimating the sizes and the prevalence in these groups. It is important to consider that many populations are not sufficiently captured in general population surveys but may contribute considerably to the total burden of HCV. Modeling studies from the UK and the USA suggest that the majority of people living with chronic HCV are either current or former PWID—with so-called “never injectors” contributing much less to the total burden of HCV (estimates from the UK suggest only around 15%) (31, 33–36). However, the epidemiology varies across Europe, with iatrogenic transmission an important driver of infection in some countries and non-PWID groups, such as migrants and MSM, affected in other countries (5, 37).

In conclusion, an evidence-based technical protocol for undertaking HCV prevalence surveys in the general population reflecting the different needs, resources and epidemiological situations across Europe has been developed and found useful through piloting (15). This technical protocol will help support EU/EEA countries in estimating their national viral hepatitis burden.

## The Sphere-C Expert Group

Laurie Barker (lub2@cdc.gov), Cecile Brouard (Cecile.BROUARD@santepubliquefrance.fr), Ana Maria Avellon Calvo (aavellon@isciii.es), Isabelle Giraudon (Isabelle.giraudon@emcdda.europa.eu), Antje Gösswald (GoesswaldA@rki.de), Susan Hahné (Susan.Hahne@rivm.nl), Greet Hendrickx (greet.hendrickx@uantwerpen.be), Vivian Hope (V.D.Hope@ljmu.ac.uk), Sharon Hutchinson (sharon.hutchinson2@nhs.net), Ana Kasradze (anakasradze@gmail.com), Anda Kivite-Urtane (anda.kivite-urtane@rsu.lv), Fiona van der Klis (fiona.van.der.klis@rivm.nl), Karine Lacombe (karine.lacombe2@aphp.fr), Angelica Maineri (a.m.maineri@tilburguniversity.edu), Ulrich Marcus (MarcusU@rki.de), Antons Mozalevskis (mozalevskisa@who.int), Gaetan Muyldermans (Gaetan.muyldermans@sciensano.be), Francesco Negro (Francesco.Negro@hcuge.ch), Odette Popovici (odette.popovici@insp.gov.ro), Magdalena Rosinska (mrosinska@pzh.gov.pl), Oana Sandulescu (oana.sandulescu@umfcd.ro), Martin Schlaud (SchlaudM@rki.de), Thomas Seyler (Thomas.Seyler@emcdda.europa.eu), Vana Sypsa (vsipsa@med.uoa.gr), Lara Tavoschi (lara.tavoschi@unipi.it), Lelia Thornton (lelia.thornton@hse.ie), Hanna Tolonen (hanna.tolonen@thl.fi), Giota Touloumi (gtouloum@med.uoa.gr), Adriana Vince (avince@bfm.hr).

## Data Availability Statement

The data analysed in this study is subject to the following licences/restrictions: The raw data supporting the conclusions of this article will be made available by the authors, without undue reservation. Requests to access these datasets should be directed to sperle-heupeli@rki.de.

## Ethics Statement

The studies involving human participants were reviewed and approved by the local ethics committees in the pilot countries. The ethics committee at the Regional Health Inspectorate in Stara Zagora, Bulgaria and the Ethics Committee at the Istituto Superiore di Sanità in Rome Italy. In Finland, ethical approval was already provided for the large population based study FinHealth2017 and the informed consent formed covered HCV, and therefore it was not needed for the nested survey. The patients/participants provided their written informed consent to participate in this study.

## Author Contributions

RZ, SN, MG, ED, and AA-G conceptualised the SPHERE-C project. RZ supervised the project. IS, SN, RZ, and MG drafted the SPHERE-C protocol. HB-K, RB, AC, EK, KL, ZN, TP, ES, and ST prepared and implemented the pilot surveys in Finland, Italy, and Bulgaria and collected and interpreted the data. IS drafted the manuscript. All authors critically revised the manuscript and approved the final version.

## Conflict of Interest

The authors declare that the research was conducted in the absence of any commercial or financial relationships that could be construed as a potential conflict of interest.

## Footnotes

^1^Available online at: http://www.cuore.iss.it/eng/factors/HES2018-2019.asp.
